# Biogenic hydroxyapatite-zinc oxide nanocomposites: A synergistic strategy for antibacterial and osteoconductive coatings on orthopedic implants

**DOI:** 10.1016/j.heliyon.2025.e42929

**Published:** 2025-02-22

**Authors:** Mohaddeseh Fatemi, Zohreh Bahrami, Marjan Bahraminasab, Farideh Nabizadeh Chianeh

**Affiliations:** a Department of Nanotechnology, Faculty of New Sciences and Technologies, Semnan University, Semnan, Iran; b Department of Tissue Engineering and Applied Cell Sciences, School of Medicine, Semnan University of Medical Sciences, Semnan, Iran; c Department of Chemistry, Faculty of Chemistry, Semnan University, Semnan, Iran

**Keywords:** Electrophoretic deposition technique, HAp-ZnO nanocomposite coatings, Antibacterial properties, Bioactivity, Ti6Al4V implants

## Abstract

Improving the biointegration of orthopedic implants with surrounding tissues is a key focus in biomaterials science. However, surfaces that enhance osteointegration may also promote bacterial colonization, leading to infections. Therefore, it is essential to balance the need for bacterial resistance with the promotion of cell attachment for osteointegration, particularly for titanium and its alloys. This investigation aimed to study the feasibility of co-depositing natural-source synthesized hydroxyapatite (HAp) nanoparticles and zinc oxide (ZnO) particles using the electrophoretic deposition technique. The goal was to create a multifunctional surface that could simultaneously accelerate bone growth and reduce the risk of infection. Five groups of coatings were prepared with different weight percentages of HAp and ZnO: 100:0, 75:25, 50:50, 25:75, and 0:100. The co-deposition was conducted on Ti6Al4V sheets for 5 min at a voltage of 90 V. The microstructure and phase composition of the synthesized powders were first analyzed. Then, the properties of the co-deposited coatings were evaluated through bioactivity and antibacterial tests. The microstructural investigation revealed crack-free coatings with a uniform distribution of particles. Comparing the antibacterial results among different groups indicated that the 50:50, 25:75 and 0:100 HAp/ZnO samples exhibited antibacterial activity against both *Escherichia coli* and *Staphylococcus aureus*. In contrast, the 100:0 and 75:25 samples showed no activity against *Escherichia coli* and negligible activity against *Staphylococcus aureus*. Additionally, all coatings demonstrated bonelike apatite formation on their surfaces in simulated body fluid, confirming their in vitro bioactivity, which was highest for 100:0 sample. The results also confirmed a synergistic effect, where HAp contributed to antibacterial properties and ZnO enhanced bioactivity in the co-deposited samples.

## 1 Introduction

Musculoskeletal disorders are the second leading cause of disability, currently impacting more than 1.7 billion people worldwide [1]. In today's globalized and highly competitive environment, increased physical activity and modern lifestyle often contribute to the development of musculoskeletal disorders, particularly among younger individuals [1]. Knee osteoarthritis (OA) is also a significant cause of disability and functional limitations, impacting millions of older adults globally. Total knee arthroplasty (TKA) is widely recognized as an effective surgical treatment for end-stage knee OA [2]. In the UK alone, over 100,000 TKAs are performed annually [3]. Projections suggest that the demand for total knee arthroplasty will rise dramatically; by 2030, the number of procedures in the United States is expected to increase by 673 %, in the UK by 173 %, and in Wales by 332 % [4]. As a result, bone and orthopedic implants have become crucial for treating damaged and fractured bones over the past decades.

Enhancing the biointegration of orthopedic implants with surrounding tissues is widely accepted as a key objective in biomaterials science [5]. Osteointegration is dependent on two processes: (i) mechanical interdigitation, which guarantees that the implant is primarily fixed to the bone following surgery; and (ii) surface-level cellular interactions, which facilitate osteoconduction, osteoinduction, and healing in the initial 3–4 months. Both procedures are essential for the best possible clinical result [6]. Nevertheless, implant surfaces that facilitate osteointegration might also encourage bacterial cell colonization, which represents a major concern. Biomaterial infection and the ensuing biofilm formation can have catastrophic consequences and lower patient's quality of life [5]. This increased vulnerability is related to the fact that the immune system's efficacy is locally reduced by the presence of a foreign body (e.g., a metallic implant) and by the tendency of bacteria to adhere to solid substrates [7]. Thus, the adherent bacteria attach to the implant's surface, grow, and multiply to form a biofilm [8]. The current approach to address this problem is through targeted antibiotic delivery. However, controlled drug delivery at the specific infection site is challenging, which can be addressed by using antimicrobial prosthetic material that prevents the infection. Infection prevention is both economical and safer than any treatment method including implant removal with surgical intervention [1]. In addition, Biofilms are highly resistant to the immune system and conventional drugs, such as antibiotics, and may also spread and infect other tissues [9]. On the other hand, attempts to prevent bacterial colonization could make biomaterial–tissue integration more complex. Therefore, biomaterial surfaces that inhibit bacterial attachment while maintaining cell functions are ideal for ensuring optimal medical implants' long-term functionality, and this is a crucial strategy to improve the outcome of implants' surgical procedures, especially for implants based on titanium (Ti) and its alloys [5,10]. Implants with such a multifunctional potential would accelerate osteointegration and healing while minimizing the risk of early/late infections.

Because of its close resemblance to the mineral part of the bone and teeth, hydroxyapatite (HAp) is the most popular bioceramic, especially in orthopedics [11]. It has a unique ability to enhance biochemical interactions with biological systems, promoting the host bone's regeneration [12,13]; but due to its poor mechanical properties, such as brittleness, low impact resistance, this material is usually applied as a coating on metallic supports, such as Ti6Al4V implants [14]. However, for many HAp-based implants, the absence of bactericidal properties causes prosthetic infection. Combining antibacterial agents with HAp coatings is one of the most effective approaches to prevent infection [15,16]. Although silver (Ag) is often considered an adequate antibacterial agent for these applications, it is not an essential element in biological systems, and its release from an implant's surface doesn't provide specific benefits to the human body and the released amounts of Ag ions exceeding a certain limit are known to be cytotoxic [10,17]. Accordingly, regarding the long-term safety of patients, essential elements possessing antibacterial properties are preferable [18].

Zinc (Zn) is an essential mineral for biological processes such as DNA synthesis, enzyme activity, and cellular metabolism [19]. Furthermore, the incorporation of Zn in an implant material promotes the proliferation and differentiation of osteoblast cells, leading to enhanced osteogenesis [20]. Thus, adding Zn to HAp coatings is expected to improve bone formation on a medical implant [21]. Ionic zinc [Zn (II)] is currently known as “the calcium of the twenty-first century” due to growing recognition of its significant functional roles in the physiological and biological systems [22]. Zn ions also exhibit antibacterial efficacy and zinc oxide (ZnO), the widely used antimicrobial agent [23], has a significant impact on biomedical implants as a coating [24]. The antibacterial activity of ZnO has been suggested to be greatly affected by the morphology [25], structure, and size where ZnO nanoparticles (NPs) showed an enhanced biocidal effect [14,26]. In fact, the presence of ZnO NPs in the coating lead to antibacterial properties [27]. The development of mixed coatings containing both HAp and ZnO particles is a promising way to reach the goals of creating bioactivity and preventing infection. However, studies on these kinds of coatings are limited and only one property at a time has been reported mostly. For example, establishing HA-ZnO composite coatings containing various concentrations of ZnO NPs on NiTi superelastic alloy via pulsed electrochemical deposition (PED) technique was considered by Khalil-Allafi et al. [28]. They investigated the bioactivity of the obtained coatings and found that the cauliflower-like apatite morphology was observed for all the samples. In the work of Sabzi et al. [29], the ceramic combination of HAp and ZnO with a weight ratio of 50:50 was deposited on NiTi shape memory alloy by the Electrophoretic Deposition (EPD) technique. The formation of biological apatite precipitates on the surface of the nanostructured coatings after 12 days of immersion in SBF was reported. Ohtsu et al. [10] designed an antibacterial HAp coating that exploits the contact-killing capabilities of ZnO. The coating was deposited on a Ti substrate using the spin coating technique and showed excellent antibacterial efficacy against *Escherichia coli* (*E. coli*) and *Staphylococcus* (*S.*) epidermidis strains. In another attempt, a novel system using ZnO and HAp NPs was described by Memarzadeh et al. as a coating material to inhibit bacterial adhesion [30]. To do this, the Electrohydrodynamic atomization (EHDA) technique was employed to deposit different mixtures of nanoscale ZnO and HAp onto the surface of glass substrates. The coatings' antimicrobial activity against *S. aureus* was determined and the results indicated that the coatings containing 100 % ZnO and 75 % ZnO-25 % HAp had significant antimicrobial activity. Regarding the simultaneous bioactive and antibacterial HAp-ZnO coatings, Mandler et al. [14] fabricated ZnO-HAp nanocomposite coatings via the EPD technique. In their work, HAp and ZnO NPs were synthesized separately, dispersed in 2-propanol simultaneously, and co-electrophoretically deposited onto Ti substrates under different potentials. They controlled the coatings' composition by tuning the applied potential and alternating the HAp to ZnO ratio in the dispersion. Bioactivity and antibacterial tests showed excellent performance by enhancing the coatings' mineralization and successful eradication of *E. coli* bacteria. In their research, Yasir et al. [31] performed the antibacterial assessment and in vitro bioactivity tests of HAp-ZnO coatings deposited on an SS 316 substrate via the EPD technique. FTIR patterns confirmed the presence of HAp-ZnO compositions. The observed improvements in the inhibition zone of the coatings and their bioactive nature jointly reflected the suitability of HA-ZnO coatings for bioimplant applications. Wang et al. [32] reported that a stable HAp-ZnO nanocoating on Ti can be beneficial for both anti-infection and osteogenic purposes. The bioactivity test showed that the composite coating could induce the apatite's formation. The resulting apatite was in a neat arrangement and preferentially grew along the (002) crystal plane, indicating favorable bioactivity. Antibacterial tests also revealed the anti-infection property of composite coating against *E. coli* and *S. aureus.* Zhou et al. [33] coated nano-HA-ZnO on a biodegradable Mg-Zn-Ca substrate via the hydrothermal method. Results showed that the coated implant was more biologically active, and due to the presence of ZnO in the coating, the in vitro antibacterial rate of the coated substrate was close to 100 %. Table 1 represents a summary of the above-mentioned studies.

**Table 1:** Summary of recent studies on HAp-ZnO composite coatings.

Substrate	Coating Technique	HAp Synthesis Source	ZnO Synthesis Source	Bioactivity Investigation	Antibacterial Investigation	Reference No.
NiTi superelastic alloy	PED	Chem.^a^	Chem.	✓	–	[28]
NiTi shape memory alloy	EPD	Chem.	Chem.	✓	–	[29]
Ti	Spin Coating			–	✓	[10]
Glass	EHDA			–	✓	[30]
Ti	EPD	Chem.	Chem.	✓	✓	[14]
SS 316	EPD	Chem.	Chem.	✓	✓	[31]
Ti	PED	Chem.	Chem.	✓	✓	[32]
Mg-Zn-Ca	Hydrothermal	Chem.	Chem.	✓	✓	[33]
Present Study	EPD	Natural	Natural	✓	✓	–

a Chem. stands for the word “Chemical”.

**Fig. 1:**
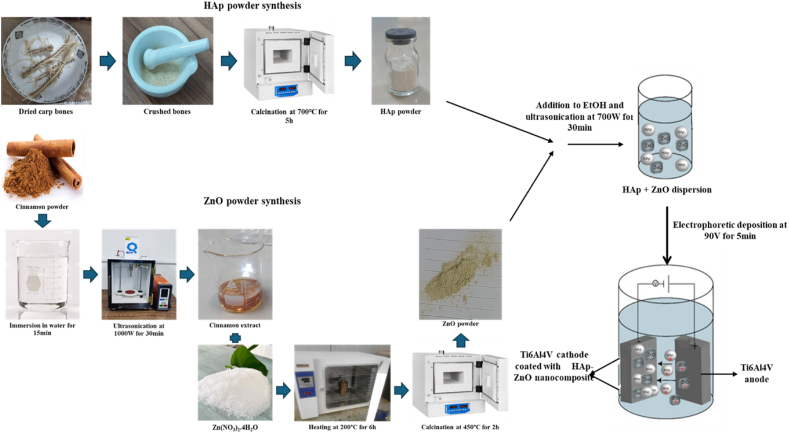
Schematic illustration of the synthesis process and formation of HAp-ZnO nanocomposite.

As shown in Table 1, there has been a limited number of studies that investigated the simultaneous antibacterial and bioactive properties of HAp and ZnO structures used as coatings. Furthermore, the HAp and ZnO particles examined in these studies were all chemically synthesized. Additionally, there is a lack of clear comparisons between samples with varying weight percentages of HAp to ZnO and their effects on coating properties. Therefore, the main novelty of the present study lies in the application-oriented synthesis of HAp and ZnO particles using carp backbone and cinnamon wood extract, respectively. This approach aims to co-deposit these particles as a coating on Ti6Al4V sheets and to evaluate their concurrent antibacterial and bioactive properties as potential candidates for orthopedic implant coatings. Fig. 1 represents the schematic illustration of the synthesis process and formation of HAp-ZnO nanocomposite.

## 2 Materials and methods

### 2.1 Synthesis of HAp NPs using carp fish backbone

Synthesis of carp bone-derived HAp was implemented according to the previous work of Bahraminasab et al. [34]. Briefly, waste carp bones were collected. The flesh parts and macroscopic adhering tissues were removed. Then, the bones were washed and boiled in water at 100 °C for about 90min to clean all debris and fat. Next, the cleansed carp bones were dried at room temperature overnight. Thereafter, the bones were crushed into very small pieces and calcified at 700 °C for 5h with a heating rate of ∼5 °C/min. Cooling was carried out slowly in the furnace to the room temperature.

### 2.2 Green synthesis of cauliflower-like ZnO particles using cinnamon extract

In this study, cinnamon extract was obtained using an ultrasonic homogenizer. First, 5g of ground cinnamon wood was placed in a beaker with 100 mL of distilled water. After immersion in water for 15min, it was subjected to 1000W ultrasonic waves for 30min. Subsequently, the obtained extract was centrifuged, and the resulting supernatant was then mixed with 0.5g of zinc nitrate tetrahydrate (Zn(NO_3_)_2_.4H_2_O) followed by stirring for 10min and drop-wise addition of nitric acid (HNO_3_ 60 %) every 30s until the zinc nitrate salt was dissolved. After stirring for 20min, the solution's pH was raised to 12 using 1M NaOH. The solution was then transferred to a Teflon-lined autoclave and kept in an oven at 200 °C for 6h. After cooling to room temperature, the resulting specimen was rinsed with water and ethanol, dried at 60 °C, and, calcined at 450 °C for 2h.

### 2.3 Suspension preparation

The EPD suspensions were prepared by adding certain amounts of HAp and ZnO powders to 20 mL of absolute ethanol (EtOH). Polyethylenimine (PEI) was employed as a dispersant, and the pH was fixed at 5.

### 2.4 EPD

The EPD cell was prepared using a 50 mL beaker equipped with a holder and Ti6Al4V sheets as both anode and cathode, respectively. The distance between the electrodes was 15 mm. The EPD voltage was 90V and the deposition process took place for 5min. After each EPD test, the samples were dried in air for 24h.

### 2.5 Characterization

The as-synthesized particles and the resulting coatings were studied using a field emission scanning electron microscope (FESEM), and their elemental analysis was measured by energy-dispersive spectroscopy (EDS). Fourier Transform Infrared Spectroscopy (FTIR) spectra were collected over the 400–4000 cm^−1^ range. The X-ray diffraction (XRD) patterns were recorded using CuKα radiation over the scanning range of 2θ = 20–70°. The Zeta potential of HAp and ZnO powders as a function of pH were analyzed using a Zeta sizer.

### 2.6 Antibacterial properties

Turbidity measurements and an agar disc diffusion test were used to investigate the coated substrates' antibacterial properties against both Gram-positive (*S. aureus*) and Gram-negative (*E. coli*) bacterial strains.

#### 2.6.1 Turbidity measurements

For this experiment, *S. aureus* and *E. coli* were cultured in liquid broth (LB) medium for 24h in a shaking incubator set at 120 rpm at 37 °C. Following the incubation, the coated samples were sterilized for 15min. under a laminar flow hood using ultraviolet (UV) light. Next, a 0.5 McFarland standard medium $(1.5\times 10^{8}$ bacteria/mL) was prepared from the cultured bacteria. In sterile conditions, 15000μL of the bacterial culture medium was added to separate tubes along with the coated samples. 15μL of the 0.5 McFarland standard concentrations of *S. aureus* and *E. coli* $(1.5\times 10^{5}$ bacteria/mL) were then added to each tube. After incubating the tubes at 37 °C for 24h, the turbidity was visually inspected, and photographs of the tubes were taken. Control samples for each bacterial strain were also prepared. To measure the optical density (OD), 100μL of all replicates of both bacterial strains were transferred to a 96-well plate, and the measurement were taken using an ELISA microplate reader at a wavelength of 600 nm. Finally, the results were statistically analyzed using GraphPad Prism Version 9 software.

#### 2.6.2 Agar well diffusion test

For the agar well diffusion test, *S. aureus* and *E. coli*. were prepared to a concentration of $1.5\times 10^{8}$ bacteria/mL, corresponding to an optical density of 0.1, and were cultured on the Mueller Hinton Agar. A total of 100 μL of sample concentrations (10 mg/mL) were introduced into each well, which had a diameter of 8 mm. The plates were then incubated at 37 °C for 24h. After the incubation period, the diameter of the inhibition zones was measured and recorded.

### 2.7 Bioactivity assessments

The in-vitro bioactivity of the coated samples was assessed by immersing them in the Simulated Body Fluid (SBF) solution for 14 days at a fixed temperature of 37 ± 0.5^o^. After the mineralization process, the samples were removed, air-dried, gently rinsed with distilled water, and air-dried again to investigate their morphology change and surface functional groups by FESEM-EDS and FTIR, respectively.

## 3 Results and discussion

### 3.1 Crystallographic characterization results

**Fig. 2:**
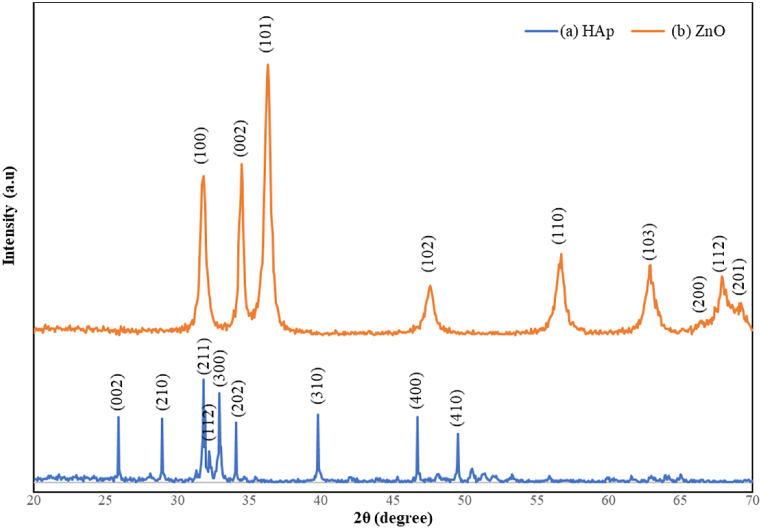
XRD patterns of the as-synthesized powders.

Fig. 2 shows the XRD patterns of the synthesized HAp and ZnO powders to investigate their crystallinity. Corresponding crystal planes are also indicated. As it is apparent in Fig. 2(a), HAp is the only phase in the powder with no impurities (JCPDS card No. 09–0432) [34,35]. Fig. 2(b) shows a single-phase hexagonal wurtzite structure (JCPDS card No. 89–7102) for ZnO [36,37]. The slightly higher intensity of the (002) diffraction peak compared with the standard JCPDS pattern suggests that ZnO particles might have preferential growth along the (002) direction, which is demonstrated through the intensity ratio of (100)/(002) planes. It has been noted earlier that the change of the relative intensity of the (100) and (002) peaks in the XRD patterns of the materials corresponds to a change in particle shape. An XRD pattern collected for bulk, isotropic ZnO gave a value of 1.17 for the (100)/(002) intensity ratio, so deviations from this value would indicate an anisotropic growth. A small (100)/(002) ratio (0.93 in this case) indicates the formation of nanorods oriented along the *c*-axis, reflecting the larger number of planes along the long axis of the rod [38].

### 3.2 Morphological results

**Fig. 3:**
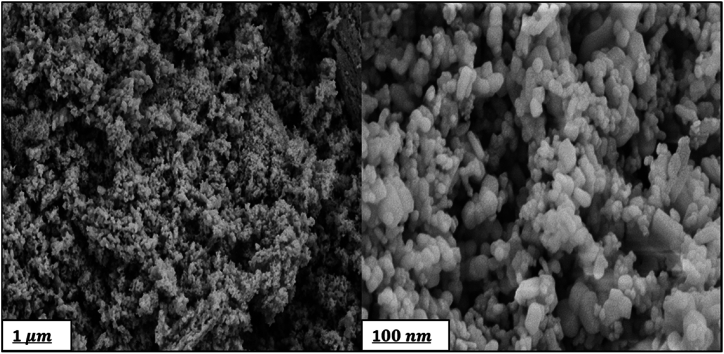
SEM micrographs of HAp NPs.

**Fig. 4:**
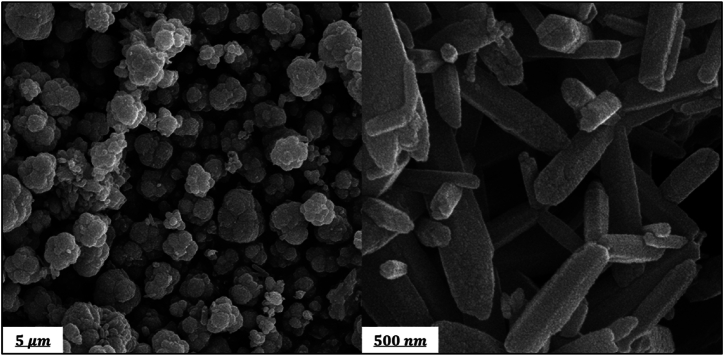
SEM micrographs of ZnO particles.

The morphology of the synthesized HAp and ZnO powders was examined by FESEM analysis depicted in Fig. 3, Fig. 4, respectively. In Fig. 3 for HAp, an interconnected porous network was observed. This nanostructure is biologically useful because it provides the body fluid with a route to pass, and the cells with pores to penetrate in and grow [34]. Fig. 4 shows a cauliflower-like structure with more central branches and rough surfaces for ZnO. Upon the synthesis, ZnO nanorods were assembled with one endpoint to form a cauliflower-like assembly. This assembly is composed of several nanocrystals extended radially from the center. Almost all assemblies had uniform sizes and presented one kind of structure [37].

### 3.3 FTIR results

**Fig. 5:**
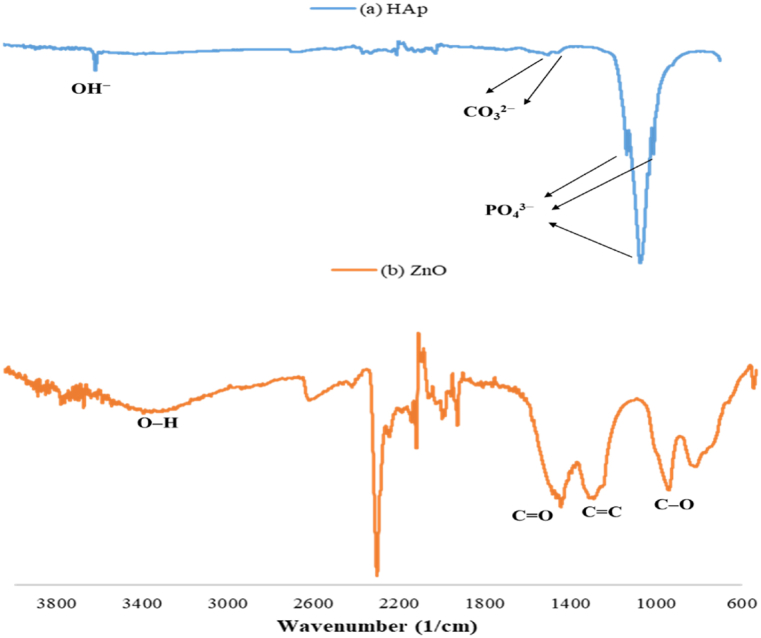
FTIR spectra of the as-synthesized powders.

FTIR spectra (Fig. 5) were recorded to obtain information about the surface functional groups of the as-synthesized powders. For HAp (Fig. 5(a)), the characteristic peaks at 1066 and 1023 cm^−1^, and the band at 962 cm^−1^ were assigned to the phosphate groups. The absorption peak at 3571 cm^−1^ was attributed to the stretching vibration of the OH^−^ (hydroxyl) group [39]. In addition, the peaks at 1457 and 1407 cm^−1^ were assigned to CO_3_^2−^ replacing the PO_4_^3−^ in HAP, thus forming carbonated HAp. The CO_3_^2−^ originated from CO_2_ in air. Studies have revealed that carbonated HAp typically shows superior bioactivity, cytocompatibility, and, if porous, osteoconduction in vivo [34,40]. For ZnO (Fig. 5(b)), the broad absorption band at 3379 cm^−1^ corresponding to the O–H stretching of free hydroxyl groups was assigned to the polyphenolic compounds of the extract. Furthermore, the absorption peak at 1520 cm^−1^ indicated the C=O stretching of ketones and carboxylic acids, whereas the peak detected at 1375 cm^−1^ revealed the C=C stretching of alkenes. Moreover, the absorption band observed at 1039 cm^−1^ corresponded to the C–O stretching of alcohols [[41], [42], [43]]. It was revealed that the C=O, C=C, and C–O groups from phytocompounds found in cinnamon were used to synthesize ZnO particles. The expected major phytocompounds that played as capping and reducing agents in the green synthesis of ZnO particles were cinnamaldehyde [44].

### 3.4 Suspension preparation and stabilization

**Fig. 6:**
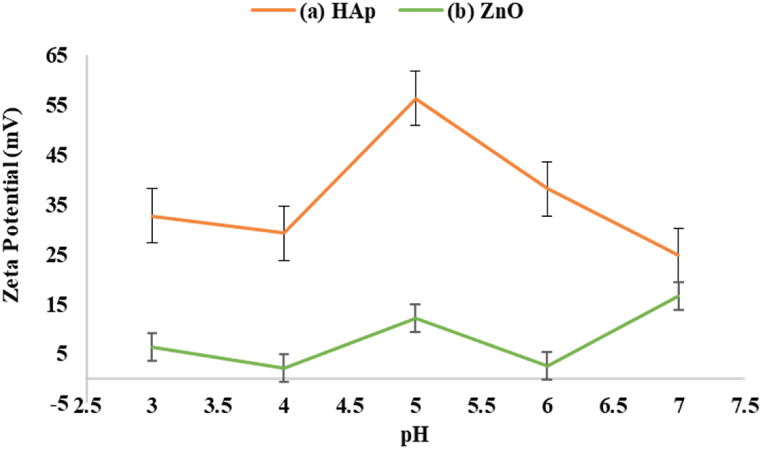
Zeta potential vs. pH measurements.

The composition and code of the samples used for making suspensions are presented in Table 2. Dispersing conditions were optimized following trial and error to obtain the best results. To do this, three steps were taken. In the first one, the optimal amount of PEI, a cationic polyelectrolyte, was determined by visual inspection of NPs stability in EtOH for 10min. Accordingly, 0.5 wt% PEI relative to HAp and ZnO powders weight was the best amount. This wt% was also used for composite samples. The second step was pH determination. So, the stability of the powders suspended in EtOH was characterized by zeta potential measurements as a function of pH (Fig. 6). As shown in Fig. 6, for HAp, maximum ζ-potential (56.3 mV) obtained at pH = 5, but its value for ZnO was 12.2 mV. Although most references state that particles with |*ξ*| around 25 mV are often stable [45,46], according to our observation, ZnO suspensions at this pH were also stable for around 5min (EPD time) and after deposition, a homogenous layer with no cracks obtained. Therefore, this pH value was chosen for the remaining EPD tests. Finally, different suspensions (HAp, ZnO, and HAp-ZnO) were prepared with a total solid loading of 5 g/L in EtOH applying high-power ultrasonication (700W for 30min.). EtOH was selected as the solvent because of the high dispersibility of both ZnO and HAp.

**Table 2:** The composition and code of the samples used for making suspensions.

Composition (Wt%)	Sample Code
HAp(100 %)	H100
HAp(75 %)-ZnO(25 %)	H75
HAp(50 %)-ZnO(50 %)	H50
HAp(25 %)-ZnO(75 %)	H25
ZnO(100 %)	H0

### 3.5 EPD obtained coatings

**Fig. 7:**
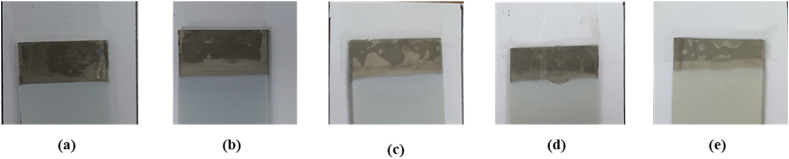
Images of the EPD-coated samples: (a) H100, (b) H75, (c) H50, (d) H25, and (e) H0.

Cathodic EPD was performed under constant voltage conditions, using 5 g/L HAp, ZnO, and HAp-ZnO nonaqueous suspensions, with 0.5 wt% PEI and pH = 5 at the voltage value of 90V for 5min. Ti6Al4V (grade 5) flat substrates were used as both anode and cathode. The distance between electrodes was 1.5 cm. As Fig. 7(a–e) indicates, all the obtained coatings are uniform with no cracks.

**Fig. 8:**
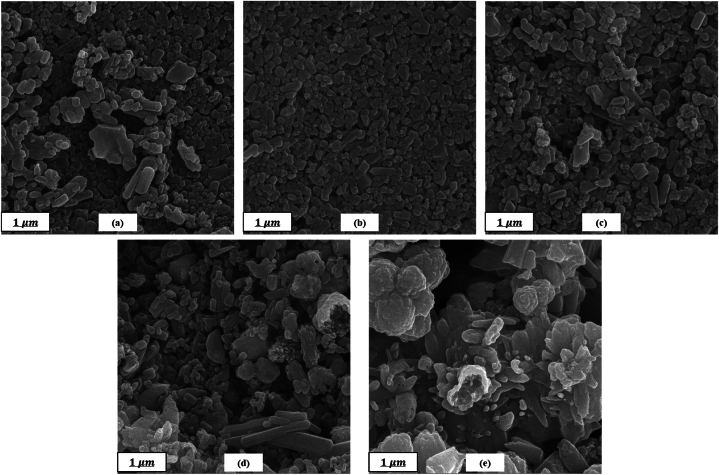
FESEM micrographs of the EPD-coated samples: (a) H100, (b) H75, (c) H50, (d) H25, and (e) H0.

**Fig. 9:**
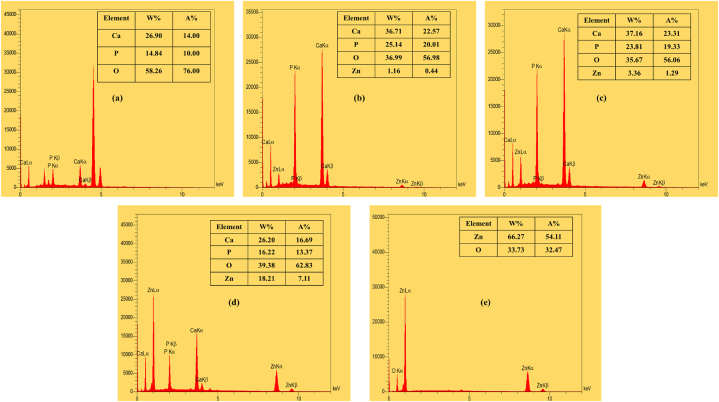
EDS spectra of the EPD-coated samples: (a) H100, (b) H75, (c) H50, (d) H25, and (e) H0.

FESEM micrographs of the coated samples are also shown in Fig. 8. All the images indicate highly dense homogenous nanostructured films. The high packing density of the films is due to the morphology and orientation of the particles. As it is obvious from the micrographs, rod-like HAp NPs are the only present morphology in the H100 sample and the dominant one in H75, but from the H50 sample cauliflowerlike coarser particles appear so that they reach the maximum amount in H0 where ZnO wt% is 100 %. These micrographs together with EDS spectra (Fig. 9) confirm the co-deposition of HAp and ZnO particles by EPD technique.

### 3.6 Antibacterial properties

**Fig. 10:**
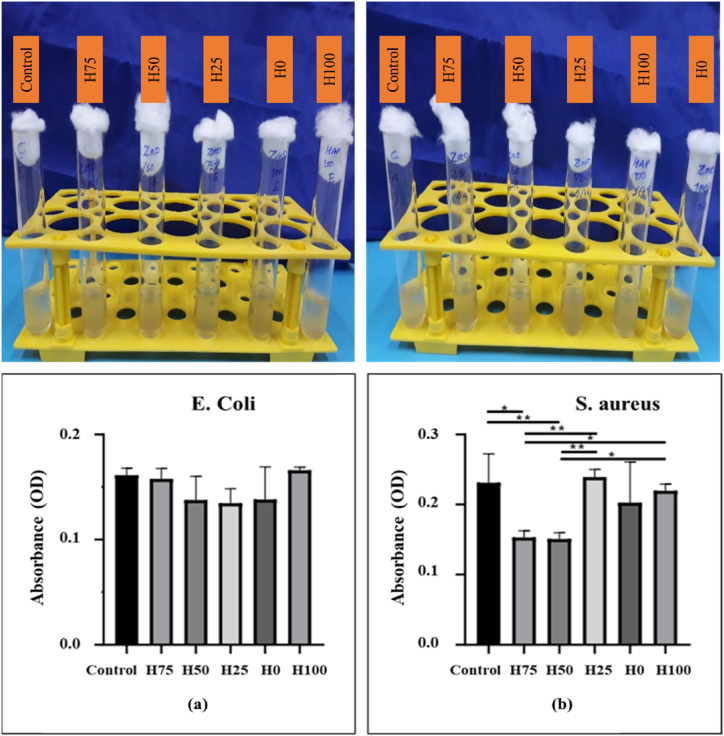
Turbidity measurement results for (a) *E. coli* and (b) *S. aureus* bacterial strains. There is no statistical difference among the groups for *E. coli* based on Tukey pair-wise comparisons. However, there are significant differences among the groups for *S. aureus* (∗P < 0.05 & ∗∗P < 0.01).

**Fig. 11:**
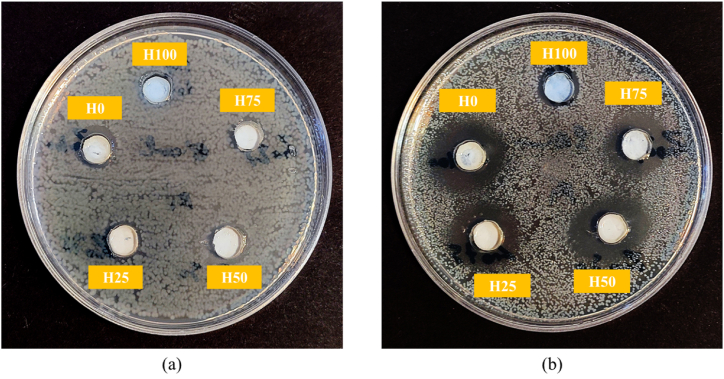
Agar disk diffusion test results for (a) *E. coli* and (b) *S. aureus* bacterial strains.

Fig. 10, Fig. 11, along with Table 3, present the results of the turbidity measurements, agar well diffusion assays, and the zones of inhibition for *E. coli* and *S. aureus* bacterial strains after 24h, respectively. As shown in Fig. 10(a) for *E. coli*, the pristine HAp (H100) and H75 samples exhibited no antimicrobial activity, while all other samples demonstrated negligible activity, which also presented the small inhibition zones depicted in Fig. 11(a) and Table 3. The incorporation of ZnO particles into the coatings (≥ 50 wt%) improved antibacterial activity against *E. coli*. In contrast, for *S. aureus*, a small inhibition zone was noted for H100 sample (Fig. 11(b) and Table 3), indicating the strain-specific antibacterial properties of this powder synthesized from carp bone. This effect may be attributed to the mechanism by which the OH groups of HAp NPs disrupt the cell wall and the structural proteins of the bacteria. The destruction of the cell wall occurs through the incorporation of N-acetyl muramic acid into mucopeptide structures, while protein denaturation caused by chemical interactions leads to the degradation of bacterial protein structure. Gram-positive bacteria possess cell walls composed of multiple layers of peptidoglycan, which are permeable, resulting in a relatively high susceptibility to antimicrobial agents [47,48]. After incorporating ZnO particles into the coatings, more distinct and larger inhibition zones were observed, indicating an enhanced antibacterial property of all the scraped coatings against this strain. This also confirms the synergistic effect of HAp nanoparticles in bacterial inhibition against *S. aureus*. It has been reported that the antibacterial properties of ZnO primarily arise from the generation of reactive oxygen species (ROS), the release of zinc ions (Zn^2+^), and contact-killing effects [10,49,50]. In the non-contact mechanism involving Zn^2+^ release, these ions adhere to the cell wall, reducing membrane permeability and negatively impacting cellular metabolism. In addition, Zn^2+^ ions can interact with several cellular components, including nucleic acids and biological enzymes, further disrupting essential metabolic processes. Specifically, Zn^2+^ binds to PsaBCA transporters in bacteria, inhibiting the uptake of manganese (Mn^2+^), which is crucial for bacterial nutrition and provides protection against oxidative stress. ROS generation constitutes another non-contact antibacterial mechanism, as these reactive species rapidly interact with cells, breaking down organic compounds. Conversely, the contact-killing mechanism depends on direct interactions between ZnO nanoparticles and bacterial cells, which can lead to the destruction of the cell wall through interactions with proteins or lipids on the bacterial surface [27,51]. When comparing the survival rates of *E. coli* and *S. aureus*, it is evident that *E. coli* displayed greater resistance across all groups. Therefore, it can be concluded that *S. aureus* was more significantly affected by all five groups of the scraped coatings as the ZnO content increases [27,52]. The differing results between Gram-positive and Gram-negative bacterial strains can be attributed to their distinct properties and morphologies. Notably, Gram-negative bacteria like *E. coli* possess an outer membrane that encases the peptidoglycan cell wall, which limits the diffusion of antibacterial materials into the cell wall and reduces the likelihood of cell wall rupture upon contact with ZnO particles. This structural feature contributes to the higher resistance of Gram-negative bacteria to antimicrobial agents compared to Gram-positive bacteria [53]. For turbidity test, statistical analyses, including one-way analysis of variance (ANOVA) and post-hoc Tukey pair-wise comparisons, were conducted to identify significant differences among the groups.

**Table 3:** Results of the zone of inhibition of the scraped coatings.

Sample Code	*E. coli* zone diameter (mm)	*S. aureus* zone diameter (mm)
H100	0	10
H75	0	15
H50	10	18
H25	10	18
H0	12	20

### 3.7 Bioactivity assessments

**Fig. 12:**
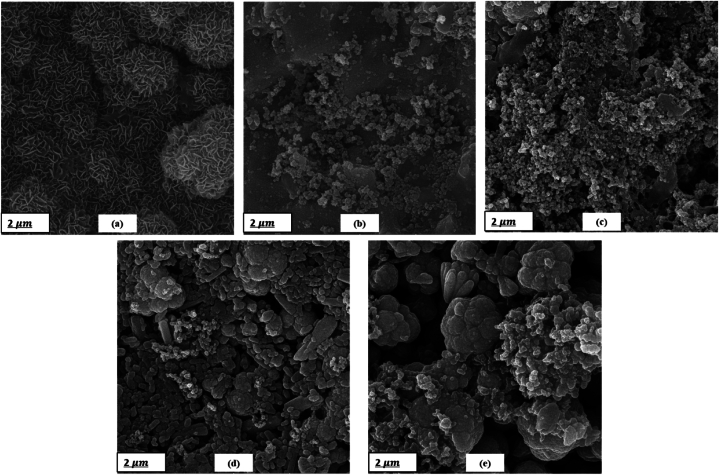
FESEM micrographs of the EPD-coated samples after immersion in SBF: (a) H100, (b) H75, (c) H50, (d) H25, and (e) H0.

**Fig. 13:**
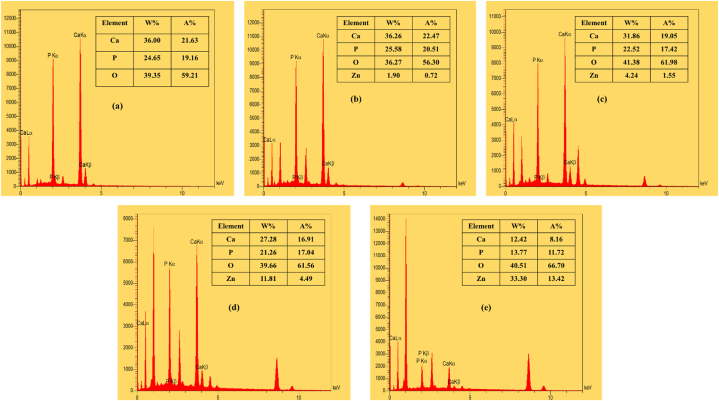
EDS spectra of the EPD-coated samples after immersion in SBF: (a) H100, (b) H75, (c) H50, (d) H25, and (e) H0.

Since the biomaterials community assumes that the formation of a HAp-like layer on the surface of a given material immersed in SBF is a proof of its bioactivity and bone-bonding ability in vivo, this issue was investigated in the present work. Fig. 12, Fig. 13 show the SEM micrographs and EDS analysis of the EPD-coated samples after bioactivity assessments, respectively. As it is evident in these figures, the surfaces of all samples indicate a degree of morphology change which is maximum for H100. Fig. 12(a), reveals the formation of calcium phosphate globular agglomerates formed by nanoscale flakes on the surface of this sample. This is expected for HAp as a bioactive species and agrees with previous reports [54,55]. The bonelike apatite formation in SBF occurs due to the high concentration of OH^−^ and PO_4_^3−^ groups on the HAp surface through interaction with the surrounding fluid's Ca^2+^ ions, leading to the formation of Ca-rich apatite. Then, the SBF's negative PO_4_^3−^ ions interact with the Ca-rich apatite surface and form Ca-poor apatite. Finally, the Ca-poor apatite on the surface of HAp gradually crystallizes into bonelike apatite, through which the HAp appears to stabilize its surface in SBF [56,57]. However, for the remaining samples (Fig. 12(b–e)) partial formation of pellet-like clusters was observed on the surface, implying the biomineralization occurrence. When the HAp-ZnO nanostructured coating is immersed in SBF, Ca^2+^ ions begin to enter the SBF medium from the coating surface. This process gradually increases the density of OH^−^ groups on the coating surface through adsorption. The OH^−^ groups then exchange with Ca^2+^ and hydrogen ions (H^+^) on the coating surface, leading to an increase in pH. The accumulation of OH^−^ ions on the coating surface is crucial for the nucleation of apatite. The dissolution of the coating surface material and the formation of the apatite layer occur simultaneously, representing a dynamic process in apatite formation. As the concentrations of PO_4_^3−^ and Ca^2+^ ions reach their maximum levels, the pH value also exhibits an increase. At this point, calcium-phosphate (Ca-P) compounds begin to form spontaneously, which results in a decrease in pH. When a balance is achieved between dissolution and precipitation, the pH value stabilizes [29]. The morphology difference of H100 with other samples is related to the presence of ZnO particles in the coatings, where zinc ions have a significant impact on the crystallization of HAp. It was suggested that partially dissolved ZnO generates Zn^2+^ ions, which are adsorbed on the active sites of HAp, and inhibit the nucleation of bonelike apatite [14]. These Zn cations will repel H^+^ ions and would not allow the local pH to increase, eventually interrupt the CaP species gathering and apatite nucleation. This event will reduce the overall number of nuclei on the surface, thereby enlarging the remained apatite at other Zn^2+^-free spots [28]. EDS spectra of Fig. 13 confirm the presence of Ca and P elements, especially in the H0 sample. This result confirms that ZnO particles are also bioactive, which is in agreement with previous studies [50,58]; however, their bioactivity is much lower than that of HAp.

**Fig. 14:**
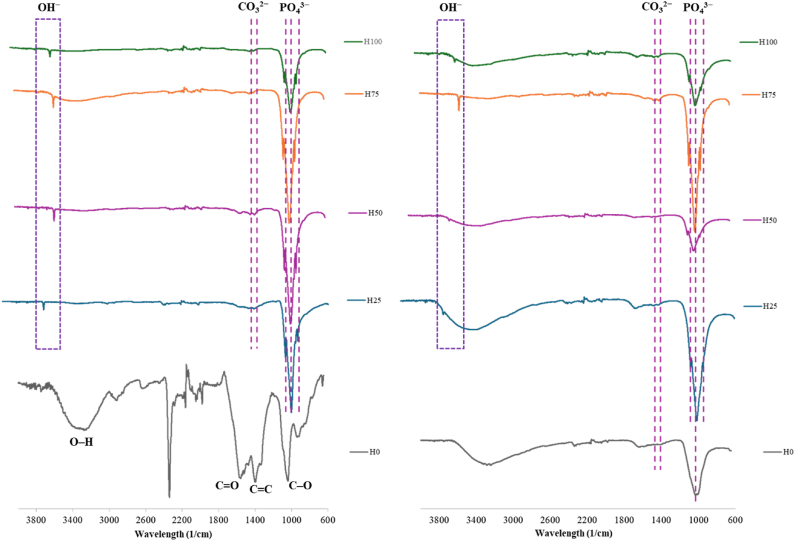
FTIR spectra of the EPD-coated samples (a) before and (b) after immersion in SBF.

Fig. 14 provides further confirmation of the presence of a HAp-like layer on the surface of all coatings, showing the FTIR spectra of the samples before and after immersion in SBF, respectively. The FTIR absorption spectrum reveals significant peaks between 900 and 1100 cm-^1^, corresponding to the PO_4_^3−^ binding group present in the structure. An absorption peak around 3571 cm^−1^ is attributed to the tensile mode of the OH^−^ group. Additionally, there are CO_3_^2−^ absorption peaks at 1457 and 1407 cm^−1^, indicating the replacement of some phosphate groups in the crystalline structure of the calcium-phosphate compounds with carbonate groups. The sharpness of all the observed peaks in the FTIR spectra indicates a high degree of crystallinity in the precipitates formed on the surface of the HAp-ZnO coatings after immersion in the SBF solution, which is consistent with previous studies [29].

## 4 Conclusion

This study focuses on the synthesis of HAp and ZnO particles derived from natural sources: carp backbone and cinnamon wood extract, respectively. The objective was to utilize these materials for the simultaneous formation of nanocomposite HAp-ZnO coatings using an electrophoretic deposition technique. Additionally, the study examined the antibacterial and bioactive properties of these coatings in vitro. Here are the main conclusions drawn from the study.

• XRD and FESEM analyses confirmed the successful synthesis of HAp NPs from carp backbone through a calcination process.
• The cinnamon wood extract, obtained through high-power ultrasonication, facilitated the formation of notable cauliflower-like ZnO structures, which were validated by XRD and FESEM analyses.
• Further analysis using FESEM and FTIR showed the successful co-deposition of HAp and ZnO particles in varying weight ratios (25:75, 50:50, and 75:25) through a one-step electrophoretic deposition technique.
• In terms of antibacterial activity, the samples with weigh ratios of 50:50, 25:75 and 0:100 HAp/ZnO exhibited antibacterial properties against both *E. coli* and *S. aureus.* Conversely, the 100:0 and 75:25 samples showed no activity against *E. coli* and negligible activity against *S. aureus.* This observation can be attributed to the distinct properties and morphologies of the two bacterial strains.
• Besides the pristine HAp coating, all other coated samples exhibited a certain degree of bioactivity, indicating their potential to enhance osteointegration in clinical applications.

Although various results have been reported, further experiments are necessary to confirm the multifunctional applications of HAp/ZnO coatings for clinical trials. Specifically, it is important to assess the ion release from the coatings to understand their antibacterial mechanisms, evaluate their in vitro toxicity, and examine their degradation in physiological solutions such as PBS to ensure their longevity. These factors will be the focus of our future research on these coatings.

## CRediT authorship contribution statement

**Mohaddeseh Fatemi:** Writing – original draft, Project administration, Investigation. **Zohreh Bahrami:** Writing – review & editing, Supervision, Methodology. **Marjan Bahraminasab:** Writing – review & editing, Supervision, Methodology. **Farideh Nabizadeh Chianeh:** Writing – review & editing, Methodology.

## Data availability statement

Data will be made available on request.

## Disclosure statement

The authors declare that they have no known competing financial interests or personal relationships that could have appeared to influence the work reported in this paper.

## Funding

The author(s) reported there is no funding associated with the work featured in this article.

## Declaration of competing interest

The authors declare that they have no known competing financial interests or personal relationships that could have appeared to influence the work reported in this paper.
